# Understanding desiccation tolerance using the resurrection plant *Boea hygrometrica* as a model system

**DOI:** 10.3389/fpls.2013.00446

**Published:** 2013-11-12

**Authors:** Jayeeta Mitra, Guanghui Xu, Bo Wang, Meijing Li, Xin Deng

**Affiliations:** ^1^Key Laboratory of Plant Resources, Institute of Botany, Chinese Academy of SciencesBeijing, China; ^2^Department of Life Science and Bioinformatics, Assam UniversitySilchar, India

**Keywords:** *Boea hygrometrica*, desiccation tolerance, gene expression regulation, resurrection plant, adaptation

## Abstract

Vegetative tissues of *Boea hygrometrica*, a member of the Gesneriaceae family, can tolerate severe water loss to desiccated state and fully recover upon rehydration. Unlike many other so called “resurrection plants,” the detached leaves of *B. hygrometrica *also possess the same level of capacity for desiccation tolerance (DT) as that of whole plant. *B. hygrometrica *is distributed widely from the tropics to northern temperate regions in East Asia and grows vigorously in areas around limestone rocks, where dehydration occurs frequently, rapidly, and profoundly. The properties of detached *B. hygrometrica *leaves and relative ease of culture have made it a useful system to study the adaptive mechanisms of DT. Extensive studies have been conducted to identify the physiological, cellular, and molecular mechanisms underlying DT in the last decade, including specific responses to water stress, such as cell wall folding and pigment-protein complex stabilizing in desiccated leaves. In this review, the insight into the structural, physiological, and biochemical, and molecular alterations that accompany the acquisition of DT in *B. hygrometrica* is described. Finally a future perspective is proposed, with an emphasis on the emerging regulatory roles of retroelements and histone modifications in the acquisition of DT, and the need of establishment of genome sequence database and high throughput techniques to identify novel regulators for fully understanding of the matrix of DT.

## INTRODUCTION

The productivity and distribution of plants are affected to a large extent by environmental conditions, due to the immobile nature of plants. A major environmental stress experienced by plants occurs during periods of water limitation, i.e., drought. Drought stress differs according to the availability of water and ranges from stochastic periods of mild water deficit to extreme water loss (desiccation). Most plants can withstand drought for a short period, via physiological and morphological changes such as stomatal closure and architecture specialization to reduce water loss and modulate water uptake, but will experience extensive cellular damage from which recovery is not possible when water content falls below 40% relative water content (RWC; Höfler et al., 1941). Only a group of plants called resurrection plants have desiccation tolerance (DT), e.g., the ability to withstand cellular water loss to 90% RWC and above (Dinakar et al., 2012; Gechev et al., 2012). These plants assume a dormant state whereby they can withstand prolonged periods of drought and resume active metabolism when water become available again. Mechanical damage, destabilization, or loss of membrane integrity, and oxidative stress related to disruption of metabolism are the major challenge for plants to survive cellular desiccation (Vicré et al., 2004a; Farrant et al., 2007). Unlike bryophytes and lichens, which can withstand rapid dehydration by the mechanism of rehydration-induced repair process, resurrection angiosperms employ more complex DT pathways that require both short and long term genetic and biochemical reactions (Farrant et al., 2009).

Desiccation tolerance is found commonly in lower plants such as lichens and bryophytes, and are absent in gymnosperms and are found rarely in pteridophytes and angiosperms (Farrant et al., 2007; Porembski, 2011). So far, about 1,300 desiccation-tolerant plants have been described, out of which only 135 species are angiosperms, which are scattered among 13 largely unrelated families (Gaff and Oliver, 2013). Among the dicotyledoneae, the Gesneriaceae contains a variety of resurrection plants. However, DT ability was reported only for several genera including *Boea*, *Ramonda*, *Paraboea*, and *Haberlea* (Müller et al., 1997; Jiang et al., 2007; Huang et al., 2012). In the two families containing the largest number of monocotyledoneae genera of desiccation-tolerant species, the desiccation-tolerant species make up only a small proportion of their genus and these genera only a small fraction of the family (Gaff and Oliver, 2013). This phenomenon suggests that DT has evolved independently from desiccation-sensitive progenitors (Oliver et al., 2000; Gaff and Oliver, 2013). Thus the DT-associated responses and the underlying mechanisms in angiosperm resurrection plants are likely diversified; some are common, while the others are species-dependent.

Gesneriaceae family contains many resurrection species. For example, *Boea hygrometrica* and *Paraboea rufescens*, that are native to the Southeast Asia, and *Haberlea rhodopensis*, *Ramonda myconi*, and *Ramonda serbica*, that distribute mainly in the Balkan Peninsula. This review will focus on the simplified research system of DT in *B. hygrometrica*, structural, physiological, and biochemical, and molecular alterations that accompany the acquisition of DT in *B. hygrometrica*. The specificity of the DT of *B. hygrometrica* will be discussed in comparison with the other resurrection species belonging to the same family that have habitats where drought is only one of the main stresses.

## A SIMPLIFIED MODEL SYSTEM TO STUDY DT USING *B.hyhgrometrica* DETACHED LEAVES

**FIGURE 1:**
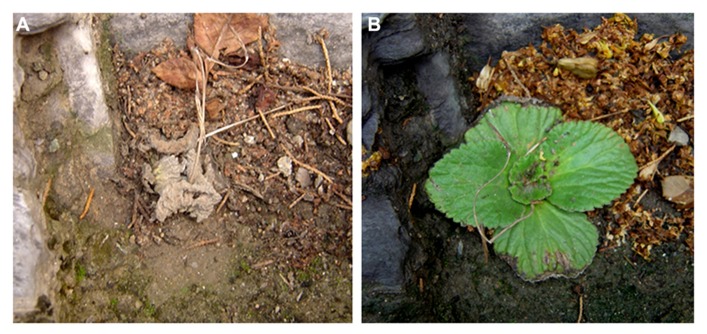
**A plant of *B. hygrometrica* at desiccated and hydrated stages.*** B. hygrometrica* plant is desiccated and shrink with a withered appearance in dry weather **(A)**, but become hydrated again after rain **(B)** in the native habitat. Photographs were taken for the same plant in Beijing Botanic Garden by Dr. Haihong Shang.

*Boea hygrometrica *is a small, perennial, and herbaceous plant belonging to the Gesneriaceae family. The species is distributed widely from the tropics to northern temperate regions in East Asia and grows vigorously in limestone rocks, where the soil is alkaline and calcium-rich, and dehydration occurs frequently, rapidly, and profoundly. *B. hygrometrica* plants are desiccated and shrink with a withered appearance in dry weather, and become hydrated again after rain in the native habitat (**Figure 1**). *B. hygrometrica* can be cultivated easily under greenhouse conditions. Seed sets with the aid of manual pollination. The seeds of *B. hygrometrica *are similar in size to *Arabidopsis thaliana* and the number of seeds in one capsule typically exceeds a hundred. The DT ability and ease of handling and maintenance has made *B. hygrometrica* a suitable model system to investigate molecular mechanism of DT.

A remarkable ability of *B. hygrometrica *is that a single detached leaf or leaf disc also possesses the same level of capacity for DT as that of whole plant, which was found only in a sub set of the resurrection plants such as *Craterostigma plantagineum*, *Myrothamnus flabellifolia, *and *Craterostigma nanum* (Gaff and Loveys, 1984; Bartels et al., 1990; Sherwin, 1995; Jiang et al., 2007). The detached leaves are useful to investigate DT, taking the advantage that these leaves are not affected by interference from developmental regulation and long*-*distance signaling from other organs during dehydration and rehydration (Jiang et al., 2007). To date, studies using this system have been conducted to characterize the architectural, physiological, cellular, and molecular mechanisms of the DT of *B. hygrometrica*, revealing dehydration responses such as cell wall folding, accumulation of raffinose oligosaccharides, late embryogenesis abundant (LEA) proteins and small heat shock proteins (sHSPs), antioxidative agents, and enzymes and stabilization of photosynthetic protein-pigment complexes (Jiang et al., 2007; Liu et al., 2009; Wang et al., 2009a; Zhang et al., 2013).

*Boea hygrometrica* survives rapid desiccation by air-drying; however, this ability is limited to natural habitats where water is periodically available. *B. hygrometrica* plants grown under well-irrigated conditions in greenhouse conditions are unable to tolerate rapid desiccation, unless pretreated with a dehydration/rehydration cycle, indicating that the slow soil drought and re-irrigation procedure is critical. The non-acclimated and acclimated plants lose water at similar rate although non-acclimated plants fail to revive after rehydration, while acclimated plants resurrect after rehydration. This characteristic has not been reported for other DT plants, yet the observation that acclimation improves drought, cold and heat tolerance had been reported in many plant species (Bayley et al., 2001; Holmstrup et al., 2002). A common view is that a period of acclimation activates stress-induced gene expression and metabolic changes which in turn are beneficial to plant survival under stress (Ahamed et al., 2012).

## THE BIOLOGICAL CHARACTERS AND STRUCTURAL ADAPTATION OF *B. hygrometrica* IN RESPONSE TO DEHYDRATION

### LEAF CURLING AND CELL WALL FOLDING DURING DEHYDRATION

Adaptive changes in leaf architecture are observed in response to periods of water deficit. These alterations are generally slower responses. For example, dehydration results in leaf shrinkage and curling toward the adaxial surface in many resurrection plants, so that the epidermis hairs on abaxial surface result in a gray-green coloration. The curling of the leaf surface and crowded epidermis hairs on the abaxial surface is considered a protective strategy against photoinhibition and reactive oxygen species (ROS) production by reducing absorption of radiation, which is followed by the accumulation of anthyocyanins and other phenolic compounds which protect against solar radiation (Farrant and Moore, 2011). This process is reversible after rehydration and related to cell wall folding. The progressive loss of water creates a tremendous amount of stress on the architecture of the plant cell, which in turn causes changes in plant cell wall polysaccharides and proteins (Jones and McQueen-Mason, 2004; Vicré et al., 2004a; Farrant et al., 2007; Moore et al., 2008; Wang et al., 2009a). The cell wall remains flexible during dehydration and becomes highly folded, which is helpful to reduce the extent of plasmolysis (Jones and McQueen-Mason, 2004; Vicré et al., 2004b; Moore et al., 2008). By cell wall folding, what is more, damage to the plasma membrane is minimized and the integrity of cell structures and the cell-to-cell communication through plasmodesmata is maintained (Neale et al., 2000; Jones and McQueen-Mason, 2004). The unbalanced folding of cell walls and shrinkage of cells in turn enables leaf curling and reversible folding (Moore et al., 2006, 2008; Farrant et al., 2007). Another process by which the plants can mitigate mechanical stress is by increased vacuolation wherein the water in vacuoles is replaced by non-aqueous substances (Oliver et al., 2011).

In *B. hygrometrica*, the contents of cell wall associated proteins and lignin were reduced in desiccated leaves (Wang et al., 2009a; Wu et al., 2009). In agreement, a gene (*BhGRP1*) encoding a cell wall structural glycine-rich protein (GRP), was isolated from the cDNA library of *B. hygrometrica *leaves dehydrated for 2h with the help of cDNA microarray approach. GRPs form a large family of heterogenous proteins that contains 60–70% of glycine residues out of the total amount of amino acid residues (Sachetto-Martins et al., 2000). There are two types of GRPs in plants. One contains an RNA-binding domain and is thought to be involved in regulation of RNA processing inside the nucleus or function similarly as that to animal cytokeratin (Mousavi and Hotta, 2005). The other class of GRPs is thought to be present in the extracellular matrix to form the structural components of plant cell walls (Sachetto-Martins et al., 2000). These GRPs are influenced by external agencies such as water, ozone stress, hormone treatment, wounding, low temperature, etc. It has also been found that water stress results in the induction of several GRP genes in both resurrection and non-resurrection plants (de Oliveira et al., 1990; Neale et al., 2000). Besides, a gene encoding a peptide highly homologous to dirigent proteins and a gene encoding germin-like proteins were identified among the dehydration-induced EST (Wang et al., 2009a; Wu et al., 2009). These proteins likely function in lignin synthesis and cell wall redox status, respectively, in turn playing a role in cell wall composition and flexibility.

### ROOT–SHOOT COMMUNICATION DURING DEHYDRATION

In the monocot resurrection plant *Sporobolus stapfianus*, evidence have shown that a root signal/s may be important for DT, according to the observation that disturbance of the root system during the drying period can disrupt the acquisition of the desiccation-tolerant state (Gaff et al., 1997). In contrast, it is unlikely that a signal from the root system is necessary for DT in aerial organs, given that the detached leaves of *B. hygrometrica* possesses the same capacity for DT as that of the whole plant. The tap root system of *B. hygrometrica *is short and weak, counting for only a small proportion of the overall biomass. However, when dehydrated as a whole plant, the roots of *B. hygrometrica* become desiccated at similar rate and exhibit similar DT ability as leaves, implying that the root system of* B. hygrometrica* maintains a congruent response to dehydration with leaves, although the signal transduction pathway is unknown for shoot–root communication.

### GROWTH REGULATION DURING DEHYDRATION

*Boea hygrometrica *plants cease growth soon after dehydration to reach a quiescent state. A heat shock factor from *B. hygrometrica*, BhHSF1, had been identified that may play a role in growth regulation in response to water limited conditions (Zhu et al., 2009). *BhHSF1* expression conferred plant stress tolerance and led to organ growth retardation, which correlated with positive regulation of drought-induced genes such as *LEAs*, *HSPs*, and *GOLS*, and negatively regulating cell division-related genes including *CDC45* and *MCM10*. Thus this factor is possibly involved in the transduction of drought signal to growth regulation, attributing to rapid acquisition of DT, and growth cessation in *B. hygrometrica*. The synergistic regulation of drought stress genes and cell cycle genes by a common transcription factor is regarded as a mechanism to integrate environmental and developmental signals and thus reprogram gene expression to ensure plant survival at the expense of growth retardation.

## PHYSIOLOGICAL AND BIOCHEMICAL PROTECTION MECHANISMS IN *B. hygrometrica* IN RESPONSE TO CELLULAR DEHYDRATION

Extensive studies have revealed several important aspects on physiological and biochemical levels that are beneficial to resurrection plant for survival under desiccation stress, for example, the control of ROS levels, the regulation of photosynthesis, and carbohydrates metabolism. Metabolic profiling of several DT plants has revealed alterations of small molecules during desiccation, such as amino acids and sugars (Oliver et al., 2011). For *B. hygrometrica*, the maintenance of photosynthetic apparatus and the protective molecules are of particular interest and discussed in the next section.

### PROTECTION OF PHOTOSYNTHETIC APPARATUS DURING DEHYDRATION

Photosynthesis is highly sensitive to the periods of water deficit. Impairment of photosynthesis, especially the leakage of electrons to O_2_ and photorespiration, results in oxidative burst in chloroplasts (Cruz de Carvalho, 2008). Photosynthesis is completely inhibited when water deficiency progresses in many resurrection plants, including *B. hygrometrica*, but in contrast to non-resurrection plants, the process is reestablished soon after rehydration (Bartels and Hussain, 2011; Porembski, 2011; Dinakar et al., 2012). The mechanism underlying photosynthesis changes is not fully understood, however the following observations have been observed in* B. hygrometrica*: (1) being homoiochlorophyllous, it retains chlorophyll contents during desiccation (Deng et al., 2003); (2) chloroplasts assume an irregular shape soon after dehydration, but the thylakoid structure remains visible under electronic microscope (Wang et al., 2009a); (3) thylakoid pigment*-*protein complexes are well conserved throughout the process of desiccation and rehydration in *B. hygrometrica *(Deng et al., 2003). The remaining of chlorophyll, thylakoid structure, and photosystem complexes contributes to rapid recovery of photosynthesis upon rehydration, but additional mechanisms will be necessary to stabilize these macromolecules and to minimize ROS accumulation, thereby protecting the plant from further damage (Farrant and Moore, 2011). In *B. hygrometrica* the carotenoid content is found increased during dehydration (Deng et al., 2003). Carotenoids play a protective role against photoinhibition. As important components of carotenoids, xanthophyll cycle pigments, are active antioxidants in chloroplast and the precursors of stress hormone abscisic acid (ABA; Georgieva et al., 2009). Furthermore, two dehydration-induced chloroplast-localized LEA proteins were found to confer drought tolerance via stabilizing photosynthesis related proteins when ectopically expressed in tobacco (Liu et al., 2009). Accordingly, carotenoids and LEA proteins are implicated to play a role in this process.

### THE ACCUMULATION OF PROTECTIVE MOLECULES IN RESPONSE TO DEHYDRATION

Antioxidant enzymes, osmolytes, and protective macromolecules accumulate to high levels in dehydrated DT plants. The accumulation of these molecules prevents accumulation of ROS, and protects membranes and proteins by forming a glass state, which reduces the metabolic rate (Oliver et al., 2000; Phillips et al., 2002; Bartels and Hussain, 2011).

#### Sugars

One of the principal osmolytes that accumulates during desiccation in DT plants, including the two European Gesneriaceae genera, *Ramonda* spp. and *Haberlea *spp., is sucrose (Ingram and Bartels, 1996; Norwood et al., 2000; Martinelli, 2008). Sucrose acts as an osmoprotectant to stabilize the structure of macromolecules to protect biological membranes (Martinelli, 2008), and may function as a signaling component to regulate carbohydrate status, growth, and energy metabolism. Besides, oligosaccharides such as raffinose and trehalose accumulate in many angiosperms during desiccation and play a prominent role by replacement and vitrification, thereby conferring cellular protection (Norwood et al., 2000; Farrant et al., 2007). Raffinose may also prevent crystallization of sucrose during drying (Müller et al., 1997). Raffinose synthesized from galactinol and sucrose by raffinose synthase protects against paraquat induced oxidative damage (Nishizawa et al., 2008). Carbohydrate reserves such as starch become the accumulate at the onset principle source for production of sucrose during prolonged dehydration when photosynthesis is suspended (Norwood et al., 2000). Another alternative source of carbon for sucrose synthesis is stachyose (Norwood et al., 2003).

In *B. hygrometrica*, dehydration-inducible genes encoding galactinol synthase and raffinose synthase have been identified (Wang et al., 2009b). The transgenic plants overexpressing *BhGoLS1* improved drought tolerance (Wang et al., 2009b). It is interesting that galactinol accumulate in *B. hygrometrica *was similar to *B. hygroscopica* and *Xerophyta viscosa*, but different from that in *Arabidopsis*. Galactinol increased to the highest levels soon after the onset of dehydration, and remain at basal levels in desiccated leaves of the resurrection species; but accumulated gradually to high levels after 14days of water stress in *Arabidopsis *(Albini et al., 1999; Peters et al., 2007; Wang et al., 2012). These observations imply that the accumulation of galactinol and raffinose may not contribute to the osmotic protection in fully desiccated leaves in at least the above-mentioned resurrection species. Hereby, the function of galactinol in DT will need further investigation.

In addition, raffinose also accumulates soon after dehydration *in B. hygrometrica*. To the contrary, raffinose (and sucrose) accumulated only when leaf RWC decreases to 25% or lower in *H. rhodopensis* (Djilianov et al., 2011). It was proposed that the initial high sucrose and raffinose concentration in *H. rhodopensis*, as revealed by the comparison with its non-DT relative *Chirita eberhardtii*, might be important for establishing the resurrection phenotype in this species (Djilianov et al., 2011). Therefore it appears that although the dynamics of raffinose accumulation may vary in individual species, the high level of raffinose in the early stage of dehydration is common to* B. hygrometrica *and *H. rhodopensis*, and the maintaining constant high levels of sucrose and raffinose might be a specific adaptation to be able to survive a very rapid dehydration in these species.

#### Protective proteins

In *B. hygrometrica *it was inferred that mechanisms exist which prevent protein aggregation and degradation (Jiang et al., 2007). Two major classes of protective proteins are sHSPs and LEA proteins, which constitute the largest group of hydrophillins in plants (Ingram and Bartels, 1996; Battaglia et al., 2008). The hydrophillins are predicted to protect proteins and macromolecules from dehydration by creating a water hydration “shell” (Oliver et al., 2011). LEA proteins were first discovered during the final stages of seed development (Dure and Chalan, 1981). Further studies have shown that LEA proteins accumulate in response to drought, freezing, salt stress, and by treatment with the phytohormone ABA (Shao et al., 2005; Tunnacliffe and Wise, 2007). Studies also revealed that LEA proteins are produced in vegetative tissues and seeds both in desiccation-sensitive and tolerant plants during drought (Piatkowski et al., 1990; Battaglia et al., 2008; Hundertmark and Hincha, 2008). Increased drought tolerance has been found in transgenic plants such as barley, *Tamarix androssowii* and *Brassica napus* which overexpress LEA genes (Xu et al., 1996; Babu et al., 2004; Park et al., 2005). LEA proteins help to minimize damages caused due to stress by functioning in the protection of membranes and proteins, and alleviate the increase in ion concentration (Ingram and Bartels, 1996; Shao et al., 2005; Tunnacliffe and Wise, 2007). In the desiccated state, LEA proteins along with sugars form a “glassy” state (Buitink and Leprince, 2004). Based on amino acid sequence homology and specific structural features LEA proteins were classified into five groups (Dure et al., 1989). So far, only two genes encoding group 4 LEA proteins had been cloned from *B. hygrometrica*. Over-expression of both *BhLEA1* and* BhLEA2* improved transgenic tobacco drought tolerance as evidenced by increased photosynthetic efficiency and membrane integrity, increased abundance of ROS scavenging enzymes such as superoxide dismutase (SOD) and proxidase (POD; Liu et al., 2009). Furthermore, chloroplastic membrane-bound proteins such as PsBO and LHCII were highly stable in drought-stressed* BhLEA1* transgenic plants, and chloroplastic stroma proteins RbcL were better conserved in drought-stressed *BhLEA2* transgenic plants, highlighting the important roles of LEA proteins in the protection of photosynthetic proteins (Liu et al., 2009).

Similarly, sHSPs protect proteins from both aggregation and dehydration by acting as molecular chaperones (Garrido et al., 2012). Ten genes encoding sHSPs were cloned from *B. hygrometrica*, among which, six cytosol-targeted sHSP coding genes were induced after desiccation and tended to remain highly abundant during rehydration (Zhang et al., 2013). It has been established that stress conditions affect cellular environment at least in part by disturbing protein folding. There are two processes to eliminate unfolded and misfolded proteins in the cells, one in the endoplasmic reticulum associated degradation (ERAD) and the other cytoplasm protein response (CPR; Mishiba et al., 2013). The finding of dehydration-inducible cytosol sHSPs coding genes indicated a role of sHSPs in the stabilization of cytosolic proteins.

#### Antioxidants and ROS scavenging enzymes

A large number of stressful conditions such as salinity, drought, highlight, toxicity, pathogens cause extra ROS (Mittler et al., 2011). The complex antioxidative defense system of plants consists of non-enzymatic and enzymatic components. Recent studies indicate that these components exist in different organelles such as chloroplasts, mitochondria, and peroxisomes (Pang and Wang, 2008). Non-enzymatic components include the major cellular redox buffers ascorbate (AsA) and glutathione (GSH) as well as tocopherol (vitamin E), carotenoids, and phenolic compounds (Mittler, 2002). Interacting with numerous cellular components, these antioxidants modulate processes from mitosis and cell elongation to senescence and cell death (De Pinto and De Gara, 2004). (Poly)-phenols together with flavonoids appear to be particularly important in resurrection plants acting as “sun screen” pigments to shade the desiccated photosynthetic apparatus, and which will help to avoid ^1^O_2_ formation (Farrant et al., 2003; Kranner and Birtic, 2005). In *R. serbica*, the content of phenolic acids is found to be unusually large in comparison with other plants (Booker and Miller, 1998; Sgherri et al., 2004). The enzymatic components comprise of several antioxidant enzymes such as SOD, catalase (CAT), guaiacol peroxidase (GPX), ascorbate peroxidase (APX), monodehydro ascorbate reductase (MDHAR), dehydroascorbate reductase (DHAR), and glutathione reductase (GR; Noctor and Foyer, 1998). These enzymes operate in different subcellular compartments and respond in concert when cells are exposed to oxidative stress. In *Craterostigma wilmsii *and *X. viscosa*, vegetative tissues show increased expression of enzymatic antioxidants genes such as APX, GR, and SOD during drying or rehydration (Ingram and Bartels, 1996; Sherwin and Farrant, 1998). Mowla et al. (2002) identified a novel stress-inducible antioxidant enzyme XvPer1 from the resurrection plant *X. viscosa*, which may function to protect nucleic acids within the nucleus against oxidative injury. In *B. hygrometrica*, a protein annotated to polyphenol oxidase precursor was identified to be induced by dehydration, along with the proteins annotated to glutathione peromidase and glutathione *S*-transferase (Jiang et al., 2007). In consistence, the content of antioxidants GSH and the activity of ROS scavenging enzyme polyphenol oxidase are increased in *B. hygrometrica* during desiccation (Jiang et al., 2007). Polyphenol activity was also found higher in desiccated leaves in *R. serbica *and *H. rhodopensis *(Jovanovic et al., 2011). In a recent study it was found out that polyphenols protect chloroplast membranes during plant desiccation and recovery by helping the membrane to mitigate oxidation damage and facilitate in starting the photosynthesis when the plant recovers (Georgieva et al., 2010).

## EVALUATION OF THE MOLECULAR MECHANISMS OF DT IN *B.hygrometrica*

The molecular mechanisms of DT in resurrection plants have been investigated since the 1990s (Bartels et al., 1990). Numerous dehydration-inducible genes have been cloned from resurrection plants as summarized by a series of reviews (Ingram and Bartels, 1996; Farrant et al., 2007; Farrant and Moore, 2011; Gechev et al., 2012; Gaff and Oliver, 2013). The advances of “omics” technologies have greatly facilitate the discovery of DT-related genes and the understanding of the molecular mechanisms of DT. Since the last century, technologies of mRNA differential display, proteomic, macroarray hybridization have been applied to study the DT mechanism in *B. hygrometrica*, which led to the discovery of many dehydration-induced genes and proteins (Deng et al., 1999; Jiang et al., 2007; Wang et al., 2009a).

Molecular studies based on these finding have revealed a general regulation module that transcription factors control the dehydration-induced expression of functional genes downstream of phytohormone-dependent and -independent signal transduction. Plants respond immediately to water stress by various kinds of physiological responses including a rapid increase in ABA concentration. Both ABA-dependent and -independent signal pathways have been revealed in the activation of gene expression. The genes encoding the protective proteins such as aldehyde dehydrogenase, heat shock factors and LEA proteins are thought to be regulated by ABA*-*directed signal pathways (Kirch et al., 2001; Deng et al., 2006; Wu et al., 2011). Dehydration-triggered gene expression is regulated by a cascade of signaling molecules such as transcription factors, calmodulins, and kinases and phosphatases. One example of hormonal signaling in *B. hygrometrica *is ABA-dependent synthesis of galactinol and raffinose family oligosaccharide (RFOs). Both *BhGoLS1 *and *BhRFS *were induced by ABA (Wang et al., 2009b; Wang et al., 2012). The activation of *BhGoLS1 *was achieved by the regulation of a dehydration and ABA-inducible WRKY transcription factor which binds to the *W-box* elements in the promoter region of *BhGoLS1* (Wang et al., 2009b).

The role of other phytohormones in DT regulation in* B. hygrometrica* has not be investigated so far, however, the study in *H. rhodopensis*, another DT species in Gesneriaceae, had revealed the active participation of jasmonic acid, salicylic acid, cytokinins, and auxins in the dehydration response (Djilianov et al., 2013). As proposed by the authors, DT appears to be strongly influenced by the earliest and very high accumulation of JA and ABA, which coincides with the accumulation of early up-regulated transcripts, and the steady high levels of SA during the whole process of desiccation (Georgieva et al., 2012; Djilianov et al., 2013). A forthcoming study on the hormone changes during dehydration and rehydration in *B. hygrometrica* will shed light on the specificity and universality in the hormone regulation of DT molecular events among species in Gesneriaceae.

Abscisic acid and calcium have been shown to interact in regulation of dehydration-induced gene expression in *B. hygrometrica*. Calcium regulates expression of a dehydration-inducible gene *BhC2DP1 *in *B. hygrometrica *(Zhang et al., 2012). *BhC2DP1* encodes a small protein with a single C2 domain protein, which is capable of binding Ca^2^^+^. Constitutive expression of *BhC2DP1 *in *Arabidopsis* resulted in an ABA-hypersensitive phenotype, which could be rescued by supplementing Ca^2^^+^-chelating agent EGTA to growth media. Thus we propose that Ca^2^^+^ is necessary for the function of BhC2DP1 in response to ABA. Consistent with this hypothesis *BhC2DP1* transcripts accumulate soon after dehydration and exposure to exogenous Ca^2^^+^.* BhC2DP1* transcription was suppressed by ABA and EGTA, however, was promoted when ABA and EGTA were simultaneously applied. This observation suggests that the transcriptional regulation of *BhC2DP1 *by ABA is Ca^2^^+^ dependent. Fine-tuning of* BhC2DP1* expression in response to drought highlights the role of exogenous calcium in the DT response.

Calcium is an essential plant macronutrient with key structural and signaling roles and it is rich in the limestone-based alkaline soil (Ji et al., 2009). Excessive Ca^2^^+^ in the rhizosphere may also cause soil alkalization and Ca^2^^+^ toxicity by preventing the germination of seeds, reducing plant growth rate and formation of tiny yellowish or gold spots in the cell walls of fruits (White and Broadley, 2003; Song et al., 2011). Interestingly, many species in Gesneriaceae family favor calcareous massifs, including both the desiccation-tolerant *B. hygrometrica*, *R. myconi*, *R. serbica, P. rufescens, H. rhodopensis*, and the desiccation intolerant species of *Chirita* spp. and *Monophyllaea* spp. (Picó and Riba, 2002; Sgherri et al., 2004; Georgieva et al., 2008; Kiew, 2009; Huang et al., 2012). The high occurrence of DT species and calciphytes in Gesneriaceae suggests a possible link between environmental calcium and DT. However, does calcium indeed involve in the regulation of DT mechanisms, how these plants limit high calcium damage, how they balances the calcium signal from high calcium stress and dehydration stress, and if the high calcium environment benefits the evolution of DT in the resurrection species of Gesneriaceae are open to question.

Likewise, dehydration of resurrection plants in their natural habitats frequently occurs in combination of different abiotic stresses, each with the potential to exacerbate the damage caused by the others. This is particularly true in the case of Gesnericear resurrection plants that have habitats where drought is not the only main problem. For example, *H. rhodopensis* plants grow in shady rock crevices on limestone at altitudes of 100–1,700 m in the central to north part in Balkan Mountains and the South in Rhodope Mountains, Ramonda serbica inhabits the shallow organo-mineral soil (pH 7.7) that develops in crevices on northern-facing carbonate rocks in the gorges in the Balkan Peninsula, and *B. hygrometrica* is native of vast area from Northern China to Southeast Asia (Jiang et al., 2007; Rakić et al., 2009; Daskalova et al., 2011; Petrova et al., 2013), growing also on limestone at altitudes of 200–1320m. In these places, the high temperature and high irradiance in summer will increase the rate of water loss and the low temperature beneath 0°C in winter could cause freezing. It has been revealed that the effects of dehydration on photochemical activity of PSII and PSI and photosynthetic oxygen evolution was stronger when desiccation was carried out at high temperature (38°C) or becomes irreversible damaged during desiccation at high light intensities (350μmolm^-^^2^s^-^^1^; Georgieva et al., 2008, 2010; Mihailova et al., 2011). However, at least the populations in the northern temperate zone are able to tolerate several cycles of desiccation and rehydration under high temperature and intensive irradiation conditions in summer, and to survive the freezing temperature in winter after gradually dehydrated to an “anhydrobiosis” (quiescent and desiccated) stage during autumn. The similarity of the habitats of these Gesneriaceae resurrection plants highlights the specificity of the DT mechanisms of these plants. Studies on genetic model plants have shown that there are multiple stress perception and signaling pathways, some of which are specific, but others may cross-talk at various steps. It has been established that cold acclimation increases plant freezing tolerance via CBF regulatory hub and overexpressing *CBF3/DREB1a*, and consequently the CBF regulon, are not only more freezing tolerant than control plants, but are also more tolerant of dehydration stress caused by either drought or high salinity (Kasuga et al., 1999; Thomashow, 2010). Influx of calcium is an important second messenger involved in activating the cold acclimation response (Doherty et al., 2009). Whether low temperature and rhizosphere calcium and alkalization have an impact on the regulation of DT mechanisms in *B. hygrometrica* and others will help to elucidate the scientific mechanisms behind the adaptive evolution and the DT acquisition of resurrection plants in Gesneriaceae.

## CONCLUSIONS AND FUTURE PERSPECTIVES

As reviewed above, the characterization of *B. hygrometrica* demonstrates that a number of effective, protective mechanisms are induced upon dehydration. A growing body of evidence has suggested that the adaptation of resurrection plants to dry environments is due to novel regulation of existing genes. Changes in gene expression result in morphological and physiological adaptations which enable survival in a desiccated state. In additional to the general regulation module of dehydration-induced gene expression on transcriptional level, there are two novel aspects that probably worth of noticing for DT-associated genetic regulation.

One of the newly recognized regulators in DT is retroelement. The role of retroelement in DT has been illustrated in the case of *CDT-1* from the resurrection species *C. plantagineum*. A series of studies have shown that the dehydration-related ABA-inducible retroelement gene *CDT-1* could direct the synthesis of a double-stranded 21bp short interfering RNA (siRNA), which opened the metabolic pathway for DT through activation of stress-responsive genes (Furini et al., 1997; Smith-Espinoza et al., 2005; Hilbricht et al., 2008). As a major type of transposons, retroelements may silence or alter expression of genes adjacent to insertion sites and generate newly acquired exons (exapted) via transposition, contribute to chromosomal rearrangements via recombination, epigenetically alter regional methylation patterns, and provide template sequences for RNA interference (Boyko and Kovalchuk, 2011; Mirouze and Paszkowski, 2011; Gutzat and Scheid, 2012). The transcriptional activation from the transposons may also trigger locus-specific siRNA mediated RNA-directed DNA methylation (Hilbricht et al., 2008; Rigal and Mathieu, 2011; Zhang and Zhu, 2011). Transposon elements can rapidly differentiate genomes within and between species has been illustrated (Piegu et al., 2006; Venner et al., 2009; He et al., 2012). A biological diversity investigation on the patterns of genetic structure of *R. myconi* populations in eight mountain regions has revealed high genetic differentiation between geographical regions (20%) and among populations within regions (9%; Dubreuil et al., 2008). To investigate the DT-associated retrotransposon elements from *B. hygrometrica* and other resurrection species will bring interesting insights into the evolution of Gesneriaceae, species differentiation, and the acquisition and regulation of DT ability in this family.

The other type of the possible novel regulators in DT may be represented in chromatin modification. Particularly, recent studies have linked histone modification with drought tolerance (Bruce et al., 2007; Kim et al., 2010). There are at least eight distinct types of modifications found on histones including the well-informed acetylation, methylation, and phosphorylation (Kouzarides, 2007). The increase of H3K4 trimethylation and H3K9 acetylation in *Arabidopsis* is associated with drought induced expression of stress response genes (Chinnusamy and Zhu, 2009). Recent research shows that H3K4me3 modification mediate the rapid induction of trainable genes in the second round of dehydration, which is a sign of stress memory in plants (Ding et al., 2012). Besides, histone modification may also display a transient up or down regulation during stress response that can also affect target gene expression (Sokol et al., 2007). Transient chromatin modifications mediate acclimation response and heritable chromatin modifications provide within-generation and trans-generational stress memory (Kouzarides, 2007). Because *B. hygrometrica* has to undergo a drought acclimation before it gains the DT ability, it may be similar with *Arabidopsis* which keeps a drought memory that brings it a more effective desiccation response. It is possible that the level of a single type of histone modification or even a combined pattern is altered and kept in *B. hygrometrica* during drought acclimation, which results in the activation of a cascade of downstream genes. Further study on the transient or long term chromatin modifications that regulate gene expression for acquisition of DT will expand the regulation frame of gene expression in resurrection plants.

Definitively, mechanisms of DT could be dissected further with the availability of a genetic system that enables gain and loss of function experiments. Currently the application of genetic approaches is limited by the inability to transform *B. hygrometrica *and the lack of genome sequence information of this species. Zhang et al. (2011) have analyzed in a systematic way representative chloroplast and mitochondrial genomes of *B. hygrometrica* and the results provide information for a better understanding of organellar genome evolution and function. Nuclear genome sequencing of *B. hygrometrica* is undergoing. Meanwhile, transformations of large genomic DNA fragments from resurrection plant as the donor to *Arabidopsis *via transformation*-*competent binary bacterial artificial chromosome (BIBAC) vectors have been employed as a genetic tool for genome*-*wide screening of functional genes, gene clusters, quantitative trait loci (QTL), transposable elements, chromatin modifications, and other genomic elements. These efforts will help to extend our understanding on the mechanisms of DT acquisition and subsequently facilitate the improvement of drought tolerance of crops and other plants with economic or ecological importance, which is highly desired in the background of global warming.

## Conflict of Interest Statement

The authors declare that the research was conducted in the absence of any commercial or financial relationships that could be construed as a potential conflict of interest.
